# Supplementary material for: Volatile Composition and Sensory Profile of Lactose-Free Kefir, and Its Acceptability by Elderly Consumers
Source: Molecules. 2022 Aug 24;27(17):5386. doi: 10.3390/molecules27175386 (PMC9457958; doi:10.3390/molecules27175386)
Supplement: Supplementary file 1 [file molecules-27-05386-s001.zip › molecules-1815172-supplementary.pdf]

Supplementary Materials

Volatile composition and sensory profile of lactose-free kefir, and its acceptability by elderly consumers

Jaroslawa Rutkowska[1,*], Agata Antoniewska-Krzeska[1], Anna Żbikowska[2], Patricia Cazón[3], Manuel Vázquez[3]

[1]Institute of Human Nutrition Sciences, Faculty of Human Nutrition, Warsaw University of Life Sciences, Nowoursynowska st.159c, 02-776 Warsaw, Poland;

[2]Institute of Food Sciences, Department of Food Technology and Assessment, Division of Fat and Oils and Food Concentrates Technology, Warsaw University of Life Sciences (WULS-SGGW), Nowoursynowska st.159c, 02-776 Warsaw, Poland

[3]Department of Analytical Chemistry, Faculty of Veterinary, University of Santiago de Compostela, 27002-Lugo, Spain

*Correspondence: jaroslawa_rutkowska@sggw.edu.pl

Table S1. Microbial count of kefir samples on the 1st day of storage.

Microbial enumeration, log CFU/ml		
Lactobacillus spp.	7.67 ± 0.18[a]	7.81 ± 0.10[a]
Lactococcus spp.	7.89 ± 0.09[b]	7.66 ± 0.07[a]
Leuconostoc spp.	5.25 ± 0.11[a]	5.45 ± 0.14[a]
LAB	7.68 ± 0.22[a]	7.93 ± 0.25[b]
Yeast	2.95 ± 0.10[a]	3.66 ± 0.12[b]

LAB - Lactic acid bacteria; CFU - Colony-forming unit

The same letters in rows indicate the lack of a significant difference at $p < 0.05$.

Table S2. Definition/explanation of the sensory attributes used in kefir sensory analysis [16,27,52,63].

Attribute	Definition
**Aroma**	
Fermented	Aroma associated with curdled milk
Creamy	Aroma associated with full-fat cream
Sour	Aroma associated with organic acids
Yeasty	Aroma associated with fermented bread dough
Sweet	Aroma associated with condensed sweetened milk
Fruity	Aroma associated with apples, peaches and other fruits
**Taste**	
Sour	Basic taste quality, typical for organic acids
Milky	Taste typical of buttermilk
Sweet	Taste stimulated by sweeteners, such as sucrose
Bitter	Taste sensation associated with caffeine
Refreshing	First cooling moment/cool and fresh effect
**Mouthfeel**	
Mouthcoat	The degree of coating on the tongue and palate during consumption
Airy	The sensation of associated with whipped cream
Astringent	The puckering sensations on the tongue and other mouth surfaces.<br>A tingling feeling on the tongue similar to carbonated mineral water.

Table S3. Absolute values of the peak area for the volatile compounds identified in kefir samples (means ± SD, n = 5)

Compounds	Rt	Lactose-free kefir	Traditional kefir
Alcohols			
Ethanol	1.67	1.37 ± 0.09	1.77 ± 0.13*
Ethanethiol	1.85	1.65 ± 0.33	1.91 ± 0.20*
1-Pentanol	4.30	0.40 ± 0.18	1.36 ± 0.03*
3-Methyl-1-butanol	4.41	nd	0.94 ± 0.01
2,3-Butanediol	6.27	0.97 ± 0.32	4.79 ± 0.48*
2-Pentanol	7.07	nd	0.28 ± 0.04
1-Hexanol	11.39	nd	0.20 ± 0.00
2-Heptanol	13.47	0.55 ± 0.00	0.22 ± 0.01*
Total		4.94 ± 0.14	11.47 ± 0.16*
Acids			
Acetic acid	2.58	22.48 ± 1.16	34.89 ± 1.20*
Butanoic acid	6.78	1.14 ± 0.22	1.02 ± 0.37
Hexanoic acid	19.37	3.21 ± 0.37	3.30 ± 0.19
Octanoic acid	26.86	0.43 ± 0.04	0.42 ± 0.05
Total		27.26 ± 0.49	39.63 ± 0.52*
Aldehydes			
Acetaldehyde	1.49	0.42 ± 0.04	0.18 ± 0.02*
3-Methylbutanal	2.81	0.98 ± 0.11	2.09 ± 0.08*
Benzaldehyde	16.80	0.07 ± 0.03	0.02 ± 0.02*
Nonanal	23.86	0.35 ± 0.01	0.10 ± 0.02*
Total		1.82 ± 0.04	2.39 ± 0.03*
Ketones			
2-Propanone	1.77	3.91 ± 0.38	7.22 ± 0.47*
2,3-Butanedione	2.19	6.34 ± 0.83	1.80 ± 0.20*
2-Butanone	2.25	6.70 ± 0.02	8.81 ± 0.56*
3-Hydroxy-2-butanone	3.73	35.32 ± 2.10	5.60 ± 0.38*
2-Heptanone	12.74	0.62 ± 0.16	0.63 ± 0.06
2-Nonanone	23.35	0.86 ± 0.00	0.29 ± 0.01*
Total		53.75 ± 0.80	24.35 ± 0.22*
Terpenes			
α-Pinene	15.14	0.16 ± 0.01	0.28 ± 0.16*
β-Pinene	17.54	0.26 ± 0.01	0.32 ± 0.18
3-Carene	19.37	0.20 ± 0.02	0.26 ± 0.16
m-Cymene	20.10	0.07 ± 0.02	0.12 ± 0.06*
D-Limonene	20.30	0.30 ± 0.00	0.42 ± 0.37*
Copaene	33.81	0.06 ± 0.01	0.10 ± 0.02
Total		1.05 ± 0.01	1.50 ± 0.12*
Other compounds			
Toluene	5.23	0.10 ± 0.03	0.13 ± 0.01
Dimethyl disulfide	4.54	0.82 ± 0.33	0.66 ± 0.17*
2-Methyltetrahydro-thiophen-3-one	18.21	0.22 ± 0.07	0.45 ± 0.22*
Total		1.14 ± 0.16	1.24 ± 0.40
NI		10.04 ± 0.18	19.42 ± 1.44

* Significantly ($p < 0.05$) different from the lactose-free kefir; NI – not identified nd - not detected; Rt - retention time
